# *Toxoplasma gondii* oocyst-driven infection in pigs, chickens and humans in northeastern China

**DOI:** 10.1186/s12917-019-2121-4

**Published:** 2019-10-25

**Authors:** Xiao-Yi Liu, Ze-Dong Wang, Saeed El-Ashram, Quan Liu

**Affiliations:** 1 College of Life Science and Engineering, Foshan University, Foshan, Guangdong Province China; 2 Military Veterinary Institute Academy of Military Medical Sciences, Changchun, Jilin Province China

**Keywords:** *Toxoplasma gondii*, Oocyst, Sporozoite, GRA7, OWP8, CCp5A, ELISA

## Abstract

**Background:**

*Toxoplasma gondii*, an intracellular apicomplexan protozoan parasite, can infect almost all warm-blooded animals. The aim of the present study was to investigate *T. gondii* oocyst-driven infection in pigs, chickens and humans in Jilin province, northeastern China.

**Results:**

The serum samples of pigs, chickens and humans were sampled and tested by indirect enzyme-linked immunosorbent assays (ELISAs) using dense granule antigen GRA7, oocyst-specific protein OWP8, and sporozoite-specific protein CCp5A, respectively. Results showed a prevalence of 16.7% by GRA7-ELISA, and 12.2% by OWP8- and CCp5A-ELISA in pigs; 10.4% by GRA7-ELISA, 13.5% by OWP8-ELISA, and 9.4% by CCp5A-ELISA in chickens; and 14.2% by GRA7-ELISA, 3.6% by OWP8-ELISA, and 3.0% by CCp5A-ELISA in humans. No significant differences were observed between *T. gondii* seroprevalence in pigs and chickens among the three antigens-based ELISAs (*P* > 0.05). However, there were significant differences between *T. gondii* seroprevalence rates in humans (*P* < 0.05). These findings demonstrated a low prevalence of *T. gondii* oocyst-driven infection in humans, a medium prevalence in pigs, and a high prevalence in chickens.

**Conclusions:**

The present study demonstrated that different oocyst-driven infection rates in different animal species, which would help to design effective strategies to prevent *T. gondii* transmission. To our knowledge, this is the first study to differentiate *T. gondii* infective forms in pigs, chickens and humans in China.

## Background

*Toxoplasma gondii*, an intracellular apicomplexan protozoan parasite, can infect nearly all warm-blooded animals, including humans and birds [1]. In human, *T. gondii* infection can be life-threatening for congenitally infected infants and immunocompromised patients [2]. The definitive hosts of *T. gondii* are members of the *Felidae* family, and the intermediate hosts include almost all warm-blooded animals. The three parasite stages, including the tachyzoites, the bradyzoites in the tissue cysts, and the sporozoites in the oocysts, are able to infect the hosts [3]. The oral route is considered the main form of postnatal transmission of toxoplasmosis in humans and animals, which may be obtained by ingestion of infective oocysts in contaminated food and water, or by ingestion of raw/undercooked meat containing tissue cysts [2, 3].

The *T. gondii* oocysts are highly resistant to freezing or disinfectants, and sporulated oocysts can survive in moist soil for months or even years, and also can be mechanically transferred from one host to the other by invertebrates [2]. Therefore, an environmental contamination with *T. gondii* oocysts is also considered as a potential source and a risk factor for warm-blooded animals, including humans. Due to the great medical importance of toxoplasmosis, numerous epidemiological surveys have been previously conducted in humans and other animals worldwide [4–10]. However, few studies of them have identified the infection sources of *T. gondii*.

Several oocyst/sporozoite-specific proteins have been identified as serological markers for oocyst-driven *T. gondii* infection. For example, the oocyst wall protein 8 (OWP8) has been confirmed to be oocyst-specific, without cross-reactivity to bradyzoite cyst wall or tachyzoite antibodies [11]. In addition, the sporozoite-specific protein, embryogenesis- related protein (ERP), has been used to differentiate oocyst- versus tissue cyst- induced infection in humans and animals [12]. Furthermore, the *Limulus* factor C-Coch-5b2-Lgl1 (LCCL) related protein, CCp5A, which is a common feature of various secreted proteins among apicomplexan parasites, expressed only in the oocyst stage of *T. gondii*, and can tell *T. gondii* oocyst infection from tissue cyst infection in vivo [13]. Another *T. gondii* marker, the dense granule antigen protein 7 (GRA7), an important secretory protein, expressed by *T. gondii* tachyzoites and bradyzoites. GRA7 locates on the surface and cytoplasmic matrix of host cells. Studies have shown that the recombinant GRA7 can perform well in the diagnostic of *T. gondii* infection in animals, including chicken, dog, cat, cattle, and human, but cannot differentiate oocyst- versus tissue cyst- induced *T. gondii* infection [14–19].

Identification of *T. gondii* infective forms can help to design effective strategies to control parasite transmission and to prevent severe complications, mainly in immunocompromised people and pregnant women. In this study, serologic differentiation of *T. gondii* infective stages in human, pig and chicken infection was conducted in Jilin province, northeastern China, using GRA7 and oocyst/sporozoite-specific proteins OWP8 and CCp5A.

## Results

### Cloning and expression of OWP8 and CCp5A

**Fig. 1:**
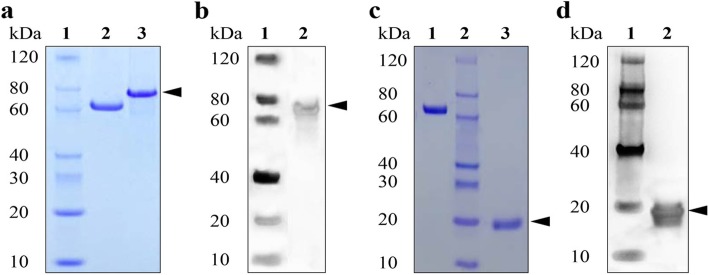
Expression of recombinant OWP8 and CCp5A of *T. gondii* in *E. coli*. **a** SDS-PAGE analysis of the purified OWP8. Lane 1: Protein molecular weight marker; Lane 2: BSA (2 μg); Lane 3: The purified OWP8 by Ni-NTA affinity chromatography (2 μg). **b** Western blot analysis of the recombinant OWP8. Lane 1: Molecular protein marker; Lane 2: The expressed OWP8. **c** The purified CCp5A analyzed by SDS-PAGE. Lane 1: BSA; Lane 2: Molecular protein marker; Lane 3: The purified CCp5A. **d** Western blot analysis of the recombinant CCp5A. Lane 1: Protein molecular weight marker; Lane 2: The expressed CCp5A; The arrows indicate the target protein

The OWP8-encoding gene was cloned into the prokaryotic vector pET30, and expressed in *E. coli* BL21 (DE3). The protein was purified by Ni-NTA affinity chromatography, showing a histidine fusion protein of approximately 65 kDa (Fig. 1a). The immunoreactivity of the expressed protein was confirmed by Western blot using the mice serum anti-His tag IgG antibodies, showing a specific signal at approximately 75 kDa (Fig. 1b).

The CCp5A, a LCCL-related protein, contains three important domains (468 bp) that was used to construct recombinant expression vector pET30-CCp5A. The protein was expressed as a histidine fusion protein of approximately 17 kDa. After analyzed with coomassie brilliant blue staining SDS-PAGE (Fig. 1c), the recombinant protein was also confirmed by Western blot using anti-His tag IgG antibodies (Fig. 1d).

### Serologic differentiation of *T. gondii* infective stages in pigs

**Fig. 2:**
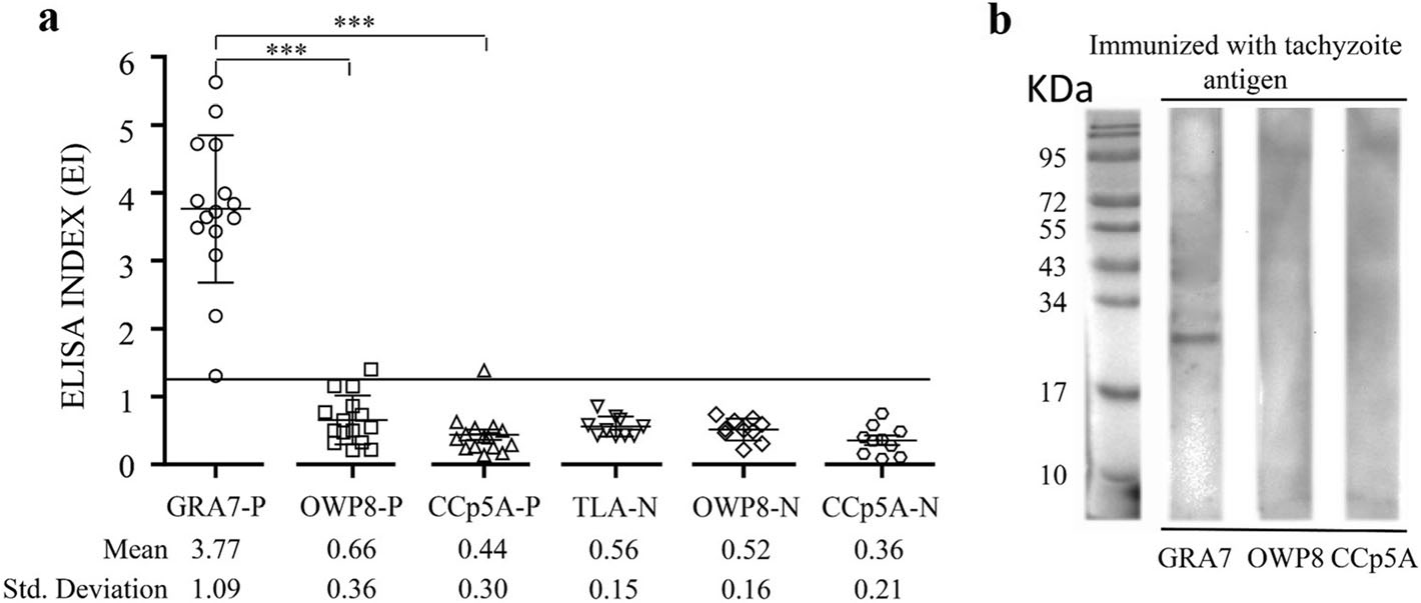
Detection of *T. gondii* tachyzoite positive and negative porcine serum samples by ELISA and Western blot using different antigens. **a** The porcine positive and negative serum samples were tested by GRA7-, OWP8-, and CCp5A-ELISAs. The dashed line indicates the cut off of the reactions. GRA7-P, OWP8-P and CCp5A-P represented the porcine positive samples for T. gondii tachyzoite infection, while GRA7-N, OWP8-N and CCp5A-N represented the porcine negative serum samples for *T. gondii* infection. **b** Western blot using serum samples from pigs experimentally infected with tachyzoites. The molecular weight markers are shown at the left side

To figure out whether OWP8 and CCp5A can serve as the protein markers of differentiating *T. gondii* infective stages, 15 *T. gondii* tachyzoite positive and 10 negative porcine serum samples were detected by GRA7-, OWP8-, and CCp5A-ELISAs, respectively. As expected, all the samples could be correctly distinguished by GRA7, with a mean ELISA index (EI, EI=OD of each sample/cut off value) of 3.77 and 0.56 for GRA7-positive and negative samples, respectively. OWP8 and CCp5A showed no reactivity for all serum samples, except for one sample had a mean EI of 0.66 and 0.52 for OWP8-positive and negative samples, and one sample with a mean EI of 0.44 and 0.36 for CCp5A-positive and negative samples, respectively (Fig. 2a). On Western blot, GRA7 presented reactivity for all the positive samples but none of the samples showed reactivity for OWP8 and CCp5A (Fig. 2b).

**Fig. 3:**
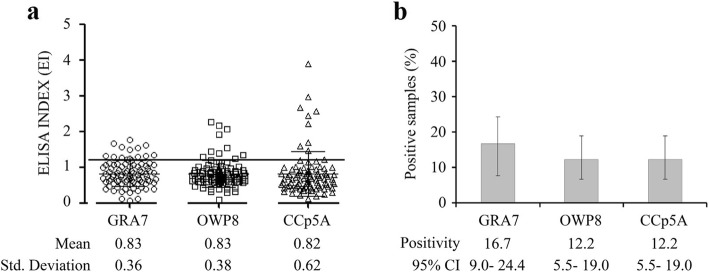
Detection of anti-*T. gondii* antibodies in serum samples of free-range pigs using different antigens. **a** The porcine serum samples were tested by GRA7-, OWP8-, and CCp5A- ELISA, The dashed line indicates the cut off of the reactions. **b** Positivity was evaluated using GRA7-, OWP8-, and CCp5A-ELISAs in serum samples from free range pigs (*n* = 90)

As shown in Table 1, of the 90 serum samples of free-range pigs, which were tested by GRA7-, OWP8-, and CCp5A-ELISA, respectively, there were 15 positive and 75 negative samples by GRA7- ELISA, 11 positive and 79 negative samples by both OWP8-, and CCp5A-ELISAs. There was no significant difference found in mean EI values (Fig. 3a). The following seroprevalence results were obtained: 16.7% (95% [CI], 9.0 to 24.4) by GRA7- ELISA, and 12.2% (95% [CI], 5.5 to 19.0) by both OWP8- and CCp5A -ELISA (Fig. 3b). Ten positive samples on both OWP8- and CCp5A-ELISA were detected among those 15 GRA7-ELISA positive samples, suggesting that *T. gondii* oocyst-driven infection rate in *T. gondii* positive porcine serum samples was 66.7% (10/15) (Table 1).

**Table 1:** Detection results of *T. gondii* antibodies by ELISA based on different antigens

Species	No. tested	No. (%) positive for GRA7	No. (%) positive for OWP8	No. (%) positive for CCp5A
Pigs	90	15 (16.7)	11 (12.2)	11 (12.2)
Chickens	96	10 (10.4)	13 (13.5	9 (9.4)
Humans	169	24 (14.2)	6 (3.6)	5 (3.0)

Samples positive for GRA7 were tested further for OWP8 and CCp5A

### Serologic differentiation of *T. gondii* infective stages in chickens

To determine sources of *T. gondii* infection in chickens, a total of 96 serum samples from free-range chickens were tested by OWP8- and CCp5A-ELISA, and compared with GRA7-ELISA. There were 10 GRA7- ELISA positive samples, while there were 13 and 9 positives in OWP8-, and CCp5A-ELISAs, respectively (Table 1).

**Fig. 4:**
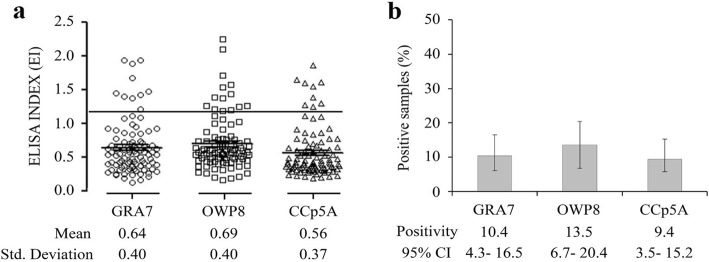
Detection of anti-*T. gondii* antibodies in free-range chicken serum samples using different antigens. **a** The chicken serum samples were tested by GRA7-, OWP8-, and CCp5A-ELISAs, and the dashed line indicates the cut off of the reactions. **b** Positivity rates were evaluated using GRA7-, OWP8-, and CCp5A-ELISAs in serum samples from free-range chickens (*n* = 96)

The IgY antibodies levels were expressed by ELISA index (EI), showing EI means of 0.64 for GRA7, 0.69 for OWP8 and 0.56 for CCp5A (Fig. 4a). The following positivity rates were obtained: 10.4% (95% CI, 4.3 to 16.5) for GRA7, 13.5% (95% CI, 6.7 to 20.4) for OWP8, and 9.4% (95% CI, 3.5 to 15.2) for CCp5A (Fig. 4b). There were no significant differences of the ELISAs and prevalence of *T. gondii* among GRA7-, OWP8-, and CCp5A-ELISAs (*P* > 0.05). Such results indicated a high *T. gondii* oocyst-driven infection rate in *T. gondii* positive chicken serum samples of 100% (10/10) by OWP8-ELISA and 80% (8/10) by CCp5A-ELISA, respectively (Table 1).

### Serologic differentiation of *T. gondii* infective stages in humans

**Fig. 5:**
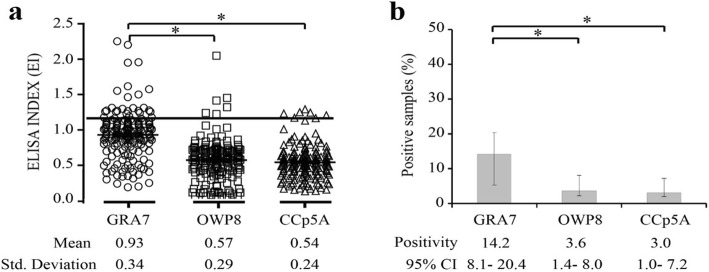
Detection of anti-*T. gondii* antibodies in human serum samples by ELISAs using different antigens. **a** The human serum samples were tested by GRA7-, OWP8-, and CCp5A-ELISAs. The dashed line indicates the cut off of the reactions. **b** Positivity rates were evaluated using GRA7-, OWP8-, and CCp5A-ELISAs in human serum samples (*n* = 169). ^∗^*P* < 0.05

To distinguish sources of *T. gondii* infection in humans, human serum samples were tested for the recombinant proteins. Of the 169 samples tested, 24 samples were positive for GRA7-ELISA, 6 for OWP8-ELISA, and 5 for CCp5A-ELISA (Table 1). Testing of IgG antibodies by ELISA showed the following EI means: 0.93 for GRA7, 0.57 for OWP8, and 0.54 for CCp5A, respectively (Fig. 5a). The following seroprevalence rates were recorded: 14.2% (95% [CI], 8.1 to 20.4) for GRA7, 3.6% (95% [CI], 1.4 to 8.0) for OWP8, and 3.0% (95% [CI], 1.0 to 7.2) for CCp5A (Fig. 5b). There were significant differences between ELISA indices and seroprevalences of *T. gondii* by GRA7-ELISA and OWP8-, CCp5A- ELISAs, respectively (*P* < 0.05). Among the GRA7-ELISA positive human serum samples, 25.0% (6/24) and 20.8% (5/24) were detected as OWP8-CCp5A-ELISAs positive, respectively, which suggested that a low *T. gondii* oocyst-driven infection rate in human beings (Table 1).

## Discussions

Previous studies have demonstrated that the oocysts/sporozoite-specific proteins OWP8 and CCp5A show specific reactivity for oocyst-infected animals, without reactivity for cysts- or tachyzoites-infected animals, while GRA7 is an important secretory protein expressed by *T. gondii* tachyzoites and bradyzoites, and is a sero-diagnostic marker for *T. gondii* infection in kinds of animals [11, 13–15, 17, 18]. In this study, *T. gondii* infection was firstly screened in different animals using GRA7-ELISA. Subsequently, the positive samples were tested by OWP8- and CCp5A-ELISAs, respectively. The results showed different oocyst-driven infection rates between 3.0 and 16.7%, ranging from a low prevalence in humans, a medium prevalence in pigs, to a high prevalence in chickens. However, further studies should be conducted on other animal species or other regions involving larger sample sizes. To our knowledge, this is the first study to differentiate infective stages of *T. gondii* in pigs, chickens and humans in China.

The specificity of oocysts/sporozoites specific proteins OWP8-, and CCp5A were also confirmed using pig serum samples immunized with tachyzoites, demonstrating that GRA7-ELISA showed a perfect detection on both positive and negative samples, while OWP8 and CCp5A showed no reactivity for all serum samples. Such results confirmed that OWP8 and CCp5A cannot detect antibodies preferentially in sera from pigs infected by *T*. gondii tachyzoite, which were further verified by Western blot.

Our study showed different oocyst-driven infection rates in different hosts of *T. gondii*, which are associated with their feeding habits or lifestyles. Consumption of raw or undercooked meat containing *T. gondii* tissue cysts is one of the main transmission routes in humans, and other infection routes also include consuming food or drink contaminated with oocysts or by accidentally ingesting oocysts from the environment, which are consistent with our findings that approximately 25% oocyst-driven infections and 75% cyst-driven infections were tested in humans in northeastern China [2, 20, 21].

Pigs and chickens are the major meat-producing animal species in China and other countries, and playing important role in the zoonotic transmission of *T. gondii* infection [21]. Free-range chickens have been considered as an important sentinel animal of soil contamination with *T. gondii* oocysts as they obtain food from the ground [22]. Thus, oocysts are considered the main infection form in chickens, as shown by a high oocyst-driven infection rates (80–100%) by different antigens. Though both OWP8 and CCp5A can be used as diagnostic markers for oocysts infection of *T. gondii*, their sensitivity may be different when applied to the clinical samples, which should be further evaluated using different animal species of *T. gondii* oocysts and cysts infections. On the other hand, it is possible for chickens to eat raw or undercooked meat containing *T. gondii* tissue cysts, though it is not the main route of infection. This study also showed pigs had a high oocyst-driven infection rates, implying that pig feed are seriously contaminated with *T. gondii* oocysts in northeastern China.

## Conclusions

The present study determined *T. gondii* oocyst-driven infection in pigs, chickens, and humans in Jilin Province, northeastern China. The differentiation of infective stages may help to design effective strategies to prevent *T. gondii* transmission in animals and humans in the studied areas.

## Methods

### Serum samples

Human blood samples were obtained from individuals who went to hospital for medical examination in Hun Chun City in Jilin Province. Animal blood samples were collected from the precaval vein of pigs and wing vein of chickens after permission was obtained from their owners in Changchun, Jilin Province, northeastern China in September, 2016. These animals were free-range in small peasant households. Sera were separated by centrifugation at 1500×g for 5 min and stored at − 80 °C until use. The *T. gondii* tachyzoite positive and negative porcine serum samples were kindly provided by Professor Xing-Quan Zhu at Lanzhou Veterinary Research Institute, Chinese Academy of Agricultural Sciences.

### Preparation of recombinant proteins GRA7, OWP8 and CCp5A

Recombinant granule antigen protein GRA7 was prepared as described elsewhere [17]. The recombinant antigens OWP8 (http://toxodb.org/toxo/; Gene ID: TGVEG_271580) and CCp5A (GenBank® accession number: EF517499) were expressed in *Escherichia coli* and purified using the standard techniques [13]. Briefly, the protein-encoding genes were cloned into pET-30a to generate recombinant plasmids pET30-OWP8 and pET30-CCp5A. The recombinant proteins were confirmed by restriction enzyme digestion and DNA sequencing analysis, and further processed for the expression of recombinant products in *E. coli* BL21 (DE3). The recombinant proteins were analyzed by SDS-PAGE using a 12% polyacrylamide gel. The electrophoretic transfer of the recombinant proteins to a 0.2 μm PVDF membrane was carried out by semi-dry transfer cell (Bio-Rad Trans-Blot® SD) at 15 V for 35 min. Blots of recombinant OWP8 and CCp5A were incubated with mouse anti-His tag sera as primary antibodies, followed by goat anti-mouse horseradish peroxidase conjugate, which were both diluted in 5% skimmed milk TBST. The recombinant proteins were purified using the Ni-NTA purification system (Qiagen, Hilden, Germany) according to the manufacturer’s protocol.

### Enzyme-linked immunosorbent assays (ELISAs)

ELISA assays were conducted to evaluate anti-*T. gondii* antibodies in pigs, chickens and humans as described elsewhere with minor modification [17]. Briefly, microplates were coated with 50 μl of GRA7 (5 μg/ml), OWP8 (5 μg/ml), or CCp5A (10 μg/ml), respectively. After overnight coating, the microplates were blocked with 100 μl PBST-5% skimmed milk for 1 h. The pig, chicken, or human sera diluted at 1:50 with 1% skimmed milk PBST was added to each well, and incubated for 1 h at 37 °C, then 50 μl horseradish peroxidase-conjugated anti-pig IgG, anti-chicken IgY, or anti-human IgG antibodies (Thermo Scientific, USA) diluted at 1:20,000 was added. After five washes, a TMB substrate solution was added and the reaction was stopped by 2 M H_2_SO_4_, and the optical density (OD) ELISA reaction was immediately measured by a microplate reader at 450 nm. Each serum sample was performed in triplicate.

The cut off value of the reaction was calculated as the mean optical density (OD) of ten *T. gondii* -negative control sera plus 3 standard deviations. The antibody titer of each sample was expressed as the ELISA index (EI, EI=OD of each sample/cut off value) as described elsewhere [13]. Samples with EI values ≥1.2 were considered positive.

### Western blot

The GRA7-, OWP8-, and CCp5A-ELISAs results of chicken serum samples were verified by Western blot as previously described [17]. Briefly, the recombinant proteins were resolved in a 12% SDS-PAGE gel and transferred to PVDF membrane. After an overnight blocking TBST-5% skimmed milk, the membranes were cut into strips and incubated with the serum samples. The membrane strips were further incubated with anti-pig IgG /anti-chicken IgY peroxidase-labeled conjugate antibodies (Thermo Scientific, USA) diluted at 1:5000. After washing, the strips were incubated with Super ECL Star (US Everbright® Inc) and the protein bands were visualized the specific antibodies. A sample was considered positive if the protein bands were observed.

### Data analysis

ELISA results were analyzed by the GraphPad Prism software version 5.0 (GraphPad Software, San Diego, CA, USA). Significant differences among IgG levels against recombinant proteins were determined by one-way ANOVA, while the differences among positivity rates were evaluated using Fisher’s exact test. Values of *P* < 0.05 were considered statistically significant.

## Data Availability

The datasets used and/or analysed during the current study are available from the corresponding author on reasonable request.

## Abbreviations

ANOVA: Analysis of variance
CCp5A: Limulus factor C-Coch-5b2-Lgl1 (LCCL) related protein
DNA: Deoxyribonucleic acid
EI: ELISA index
ELISA: Enzyme-linked immunosorbent assay
ERP: Embryogenesis- related protein
GRA7: Granule antigen protein 7
OD: Optical density
OWP8: Oocyst wall protein 8
PVDF: Polyvinylidene fluoride
SDS-PAGE: Sodium dodecylsulfate-polyacrylamide gel electrophoresis
